# Molecular Epidemiology of *Staphylococcus aureus* Skin and Soft Tissue Infections in the Lao People’s Democratic Republic

**DOI:** 10.4269/ajtmh.16-0746

**Published:** 2017-05-30

**Authors:** Alicia D. Yeap, Kate Woods, David A. B. Dance, Bruno Pichon, Sayaphet Rattanavong, Viengmon Davong, Rattanaphone Phetsouvanh, Paul N. Newton, Nandini Shetty, Angela M. Kearns

**Affiliations:** 1 Antimicrobial Resistance and Healthcare Associated Infections Reference Unit, National Infection Service, Public Health England, London, United Kingdom;; 2 Lao-Oxford-Mahosot Hospital-Wellcome Trust Research Unit, Microbiology Laboratory, Mahosot Hospital, Vientiane, Lao People’s Democratic Republic;; 3 Centre for Tropical Medicine and Global Health, University of Oxford, Oxford, United Kingdom;; 4 Faculty of Infectious and Tropical Diseases, London School of Hygiene and Tropical Medicine, London, United Kingdom

## Abstract

This is the first report of the molecular epidemiology of *Staphylococcus aureus* from skin and soft tissue infections (SSTI) in Laos. We selected a random sample of 96 *S. aureus* SSTI isolates received by the Microbiology Laboratory, Mahosot Hospital, Vientiane, between July 2012 and June 2014, including representation from seven referral hospitals. Isolates underwent susceptibility testing by Clinical and Laboratory Standards Institute methods, *spa* typing and DNA microarray analysis, with whole genome sequencing for rare lineages. Median patient age was 19.5 years (interquartile range 2–48.5 years); 52% (50) were female. Forty-three *spa* types, representing 17 lineages, were identified. Fifty-eight percent (56) of all isolates encoded Panton-Valentine leukocidin (PVL), representing six lineages: half of these patients had abscesses and three had positive blood cultures. The dominant lineage was CC121 (39; 41%); all but one isolate encoded PVL and 49% (19) were from children under five. *Staphyococcus argenteus* was identified in six (6%) patients; mostly adults > 50 years and with diabetes. Six isolates (6%) belonged to rare lineage ST2885; two possibly indicate cross-infection in a neonatal unit. One isolate from a previously undescribed lineage, ST1541, was identified. Antibiotic resistance was uncommon except for penicillin (93; 97%) and tetracycline (48; 50%). Seven (7%) isolates were methicillin-resistant *S. aureus* (MRSA), belonging to ST239-MRSA-III, CC59-MRSA-V(T) Taiwan Clone, ST2250-MRSA-IV, ST2885-MRSA-V and CC398-MRSA-V. Globally widespread CC5 and CC30 were absent. There are parallels in *S. aureus* molecular epidemiology between Laos and neighboring countries and these data highlight the prominence of PVL and suggest infiltration of MRSA clones of epidemic potential from surrounding countries.

## INTRODUCTION

*Staphylococcus aureus* is a leading cause of bacteremia in the Lao People’s Democratic Republic (Lao PDR; Laos), particularly in infants.^1^ The presence of skin infection and the local practice of warming mothers with their newborns on a “hot bed” (a bed under which hot coals are placed) for the first few weeks postpartum have been associated with *S. aureus* bacteremia in neonates.^2^

Methicillin-resistant *S. aureus* (MRSA) was first detected in Laos in 2001^3^ and appears to have been rare with only 10 cases identified between 2001 and 2011.^4^ This is in stark contrast to neighboring Thailand and Vietnam where MRSA accounts for 57–74% of healthcare-associated *S. aureus* infections and 3–30% of community-acquired *S. aureus* infections, with ST239-MRSA-III the dominant clone.^5^ In Cambodia, community-acquired MRSA (CA-MRSA) is an important cause of pediatric skin and soft tissue infections (SSTI)^6^; ST121-MRSA-V and ST834-MRSA-IV are the major CA-MRSA lineages in this country.^7^

A distinct lineage of *S. aureus* belonging to multilocus sequence type clonal complex 75 (MLST-CC75) and ST1223, now known as *S. argenteus* sp. nov., is responsible for 71% of CA-MRSA isolates causing SSTIs in remote aboriginal communities in northern Australia.^8^ It has also been found in human carriers in Cambodia^9^ and in invasive infections in northeast Thailand.^10^ Apart from lacking the characteristic golden pigment staphyloxanthin,^11^ *S. argenteus* is indistinguishable from *S. aureus* on routine laboratory testing such as coagulase and catalase.^12^

The molecular epidemiology of *S. aureus* infections in resource-limited countries of southeast Asia is poorly described^7^ and has not been studied in Laos. The majority of Lao are remote rural farmers living in close proximity to their livestock and although tourism is increasing, most Lao rarely travel abroad, giving rise to relative isolation. Laos may therefore represent an unusual epidemiological niche for *S. aureus*. This study reports the clinical, phenotypic and genotypic characteristics of 96 *S. aureus* SSTI isolates from Laos over a 2-year period.

## MATERIALS AND METHODS

### Study site.

The Microbiology Laboratory, Mahosot Hospital, Vientiane, Laos, receives samples from inpatients and outpatients at Mahosot Hospital, other hospitals within Vientiane and provincial study sites, of the Lao-Oxford-Mahosot-Wellcome Trust Research Unit, such as the hospitals in Luangnamtha, Xiengkhuang, and Saravane. Cloxacillin is the first-line treatment used for staphylococcal infections in Laos. Other antibiotic options include doxycycline, clarithromycin, ciprofloxacin, chloramphenicol, co-trimoxazole, ceftriaxone, and gentamicin. Glycopeptides, clindamycin, and linezolid are not currently available in Laos.

### Ethics.

All samples were received as routine diagnostic samples and data from stored isolates were anonymized prior to inclusion in the analysis. No additional samples were taken as part of this study.

### Sample selection.

Prospectively-collected consecutive *S. aureus* isolates from pus or swab samples taken between July 1, 2012 and June 30, 2014 and processed at the Microbiology Laboratory, Mahosot Hospital were archived. A random sample of 100 isolates, stratified to include a representative proportion from referral sites, was selected using Microsoft Excel’s RANDBETWEEN function. Two archived samples were missing and two grew only coagulase-negative staphylococci on subculture, leaving 96 isolates originating from different patients for analysis.

### Patient-related information.

Patients’ demographic and clinical characteristics were derived from information recorded on request forms.

### Organism identification and phenotypic antibiotic susceptibility testing.

Patient samples were cultured on 5% goat blood agar (Columbia Agar base, Oxoid, United Kingdom) and chocolate agar (Thayer Martin medium with Vitox, Oxoid, United Kingdom). *Staphylococcus aureus* were identified by Gram stain, catalase, latex agglutination or slide coagulase, tube coagulase and DNAse. Isolates were designated as *S. argenteus* based on *spa* typing and microarray lineage results. Antibiotic susceptibility testing was performed using disc diffusion methodology, or Etests for oxacillin and vancomycin, according to Clinical and Laboratory Standards Institute standards current at the time of primary isolation. Routinely tested antimicrobials were: penicillin, cefoxitin and/or oxacillin, erythromycin, tetracycline, gentamicin, chloramphenicol, and co-trimoxazole. Ciprofloxacin and vancomycin susceptibilities were determined for MRSA isolates. Blood cultures were performed as previously described.^1^

### Genotypic characterization.

Molecular analyses were carried out at Public Health England (PHE, Colindale, London). Staphylococcal protein A gene (*spa*) typing was performed^13^ and MLST-CC assignments were inferred by reference to the *spa* server (http://spa.ridom.de/mlst.shtml), MLST database (http://saureus.mlst.net) and in-house PHE database. Where a PCR product was not obtained, alternative primers were used (Pichon B, unpublished).

DNA microarray-based analyses were performed on all isolates using the StaphyType Kit 2.0 (Alere Technologies, GmbH, Jena, Germany) according to manufacturer’s instructions. DNA was extracted using the PurElute^™^ Bacterial Genomic Kit (Edge BioSystems, Gaithersburg, MD). Assignment scores indicate the similarity of test isolates to known strains - scores below 88% indicate a strain which has not previously been described in the database, or technical error. Despite repeat testing, seven isolates gave a low score of < 88% and were subjected to whole genome sequencing (WGS), using the Illumina HiSeq 2500 platform to determine the lineage (MLST) together with carriage of antimicrobial resistance and virulence genes as described previously.^14,15^

## RESULTS

### Patients.

Of the 96 patients, 50 (52%) were female. Median age was 19.5 years (interquartile range 2–48.5 years). Thirty-seven patients (39%) were under 5 years of age, including eight neonates (under 4 weeks of age) and five infants (1–12 months of age). Seventy-five patients were from Mahosot Hospital and the remaining 21 from seven referral hospitals, each accounting for one to six patients. Of 43 patients (45%) for whom dates of hospital admission were available, 37 (86%) had samples taken within 2 days of admission. Fifteen patients (16%) were outpatients. Thirty-three (34%) patients were noted to have abscesses, of which 28 (85%) had isolates which encoded Panton-Valentine leukocidin (PVL).

Blood cultures were taken for 44 patients (46%). Three (7%) had *S. aureus* bacteremia, two were infants and the third was 12 years of age. All three samples were taken within 2 days of hospitalization. Seven of the eight neonates had blood cultures taken but none were positive.

### Antibiotic susceptibilities.

Antibiotic susceptibilities and acquired resistance genes are displayed in Table 1. Seven (7%) patients’ isolates identified as MRSA both phenotypically (resistant to cefoxitin and/or oxacillin) and genotypically (*mecA*-positive); *mecC* was not found.

**Table 1:** Antibiotic susceptibilities and resistance genes by lineage for all isolates (*N* = 96)

	Total	Pen R	*blaZ*	Ery R	*ermA*	*ermB*	*ermC*	Tet R	*tetK*	*tetM*	Gen R	*aacA-aphD*	Chl R	*cat*	Cot R	*dfrS1*	*qacA*	Cip R
CC121-MSSA	39	39	39	3			3	14	17				4	4				
CC1-MSSA	12	11	11					3	1									
CC6-MSSA	6	6	6					1		1			2	2				
ST2250-MSSA	4	4	4					4	1									
CC1223-MSSA	1	1	1					1	1									
ST2885-MSSA	5	5	5					5	5				1					
CC88-MSSA	5	5	5					3	3									
CC398-MSSA	4	4	4	3	3			1	1									
CC188-MSSA	4	4	4	1		1		2	1		1	1	1	1				
CC97-MSSA	3	2	2					3	3				1					
CC20-MSSA	2	2	2	1			1	1	1		1	1	1	1			1	
CC15-MSSA	1	1	1					1	1									
ST834-MSSA	1	1	1					1	1									
ST2482-MSSA	1	1	1					1	1									
ST1541-MSSA	1	1	1															
ST239-MRSA-III	2	2	2	2	2			2	2	2	2	2			2			2
ST59/952-MRSA-V(T)	2	2	2	1		1		2	2				2	2				*
ST2250-MRSA-IV	1	1	1					1										*
ST2885-MRSA-V	1	1	1					1	1									*
CC398-MRSA-V	1	1	1	1	1			1	1									
Total	96	94	94	12	6	2	4	48	43	3	4	4	12	10	2	0	1	2

CC = clonal complex; ST = multilocus sequence type; MSSA = methicillin-sensitive *Staphylococcus aureus*; MRSA = methicillin-resistant *S. aureus*; R = phenotypically resistant on in-vitro susceptibility testing; Pen = penicillin; *blaZ* = beta-lactamase gene; Ery = erythromycin; *ermA-C,* rRNA methyltransferase genes associated with macrolide/lincosamide resistance; Tet = tetracycline; *tetK =* tetracycline efflux protein gene; *tetM =* ribosomal protection protein gene associated with tetracycline resistance; Gen = gentamicin; *aacA-aphD* = aminoglycoside adenyl-/phosphotransferase gene associated with resistance to gentamicin and tobramycin; Chl = chloramphenicol; *cat*, chloramphenicol acetyltransferase gene; Cot = co-trimoxazole; *dfrS1* = dihydrofolate reductase gene mediating trimethoprim resistance; *qacA*, quaternary ammonium compound/multidrug efflux protein A gene; Cip = ciprofloxacin.

*Ciprofloxacin phenotypic susceptibility testing results not available for these isolates; MSSA isolates were not routinely tested for susceptibility to ciprofloxacin.

94 of 96 isolates (98%) were penicillin-resistant and *blaZ*-positive. Tetracycline resistance was also common (48, 50%) and mainly associated with *tetK* (43, 90%), with *tetM* found in three isolates. Acquired tetracycline resistance genes were not detected in seven isolates that were phenotypically resistant, suggesting the presence of other mechanisms such as efflux pumps.

Erythromycin resistance was found in 12 isolates (13%), associated with *ermA* (6, 50%), *ermB* (2, 17%), and *ermC* (4, 33%). Chloramphenicol resistance was identified in 12 isolates (10%), mostly associated with *cat; fexA* was not detected. Only four isolates (4%) were resistant to gentamicin and *aacA-aphD* was detected in all. Resistance to co-trimoxazole was not detected phenotypically or genotypically. Of the three MRSA isolates that were tested against ciprofloxacin, resistance to this antibiotic was found in two. All seven MRSA isolates were susceptible to vancomycin.

Only one isolate carried *qac* genes (multidrug efflux pump). Genes conferring resistance to glycopeptides *(vanA, vanB, vanZ),* linezolid (*cfr*), mupirocin *(mupA)*, fusidic acid *(fusB, fusC)* and macrolides apart from *erm* (*msrA, mefA, mphC)* were not detected.

### Lineages and *spa* types.

**Figure 1.**
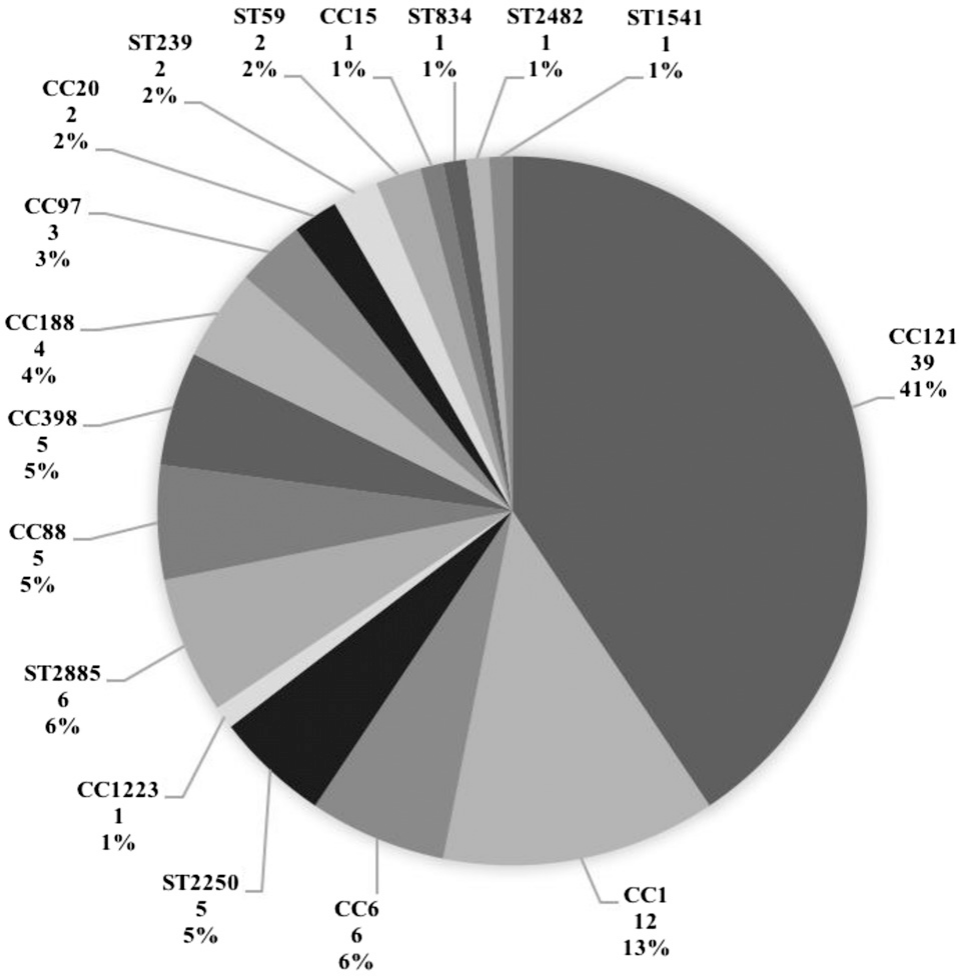
*Staphylococcus aureus* lineage distribution by microarray for all isolates (*N* = 96). CC, clonal complex; ST, multilocus sequence type; MSSA, methicillin-sensitive *Staphylococcus aureus*; MRSA, methicillin-resistant *S. aureus*. The number under each lineage label denotes the number of study isolates belonging to the lineage and the percentage denotes the representation of the lineage as a percentage of the total number of study isolates analyzed.

The distribution of lineages based on microarray is shown in Figure 1. CC121 was the predominant lineage (39; 41%), followed by CC1 (12; 13%). *Staphylococcus argenteus* lineages ST2250 and CC1223 accounted for 6% (6) of isolates. We identified six isolates (6%), including one MRSA, belonging to an uncommon lineage, ST2885, and one isolate of a previously undescribed sequence type, ST1541, which is unrelated to other lineages using eBURST analysis (http://saureus.mlst.net/eburst/).

We found 43 *spa* types belonging to 17 lineages (Supplemental Table 1). Interestingly, among the new *spa* types defined in this study, five *spa* type t13849 isolates belonging to ST2885 were identified, all from Mahosot Hospital. Three patients were neonates in the pediatric intensive care unit and single nucleotide polymorphism-based analysis of WGS data showed no variation between two isolates taken 7 months apart. There were no clear epidemiological links between patients for other *spa* types.

### Lineage-specific characteristics.

Nineteen of 39 (49%) patients with the dominant CC121 lineage were children under five. All except one of 12 patients with CC1 isolates (92%) were aged under 12 years. Among those with *S. argenteus* infections, five of six (83%) were over 50 years of age and four of six (67%) had diabetes mellitus recorded as a co-morbidity. In contrast, only 13 of the 92 patients (14%) with non-*S. argenteus* infections were noted to be diabetic. The previously undescribed ST1541 isolate originated from a 52-year-old woman who had been hospitalized for 3 days and had negative blood cultures.

The lineages found for bacteremic patients were CC121-MSSA for two and CC188-MSSA for one; all were PVL-positive and the CC188-MSSA isolate was *etA*-positive. Apart from *blaZ*-mediated penicillin resistance, all three isolates were otherwise susceptible to routinely-tested antibiotics, except for *tetK*-mediated tetracycline resistance in a CC121 isolate. No other antibiotic resistance markers were identified.

### MRSA patients and lineages.

Five (71%) patients with MRSA were female and six (86%) were adults. Two patients (29%) had been hospitalized for more than 2 days and a further patient (14%) had had recent healthcare contact. All were patients in hospitals within Vientiane, with no obvious epidemiological links between patients. One patient had recently returned from residence in Vietnam. Three (43%) had blood cultures taken, all of which were negative. Treatment information was available for four patients: initial antibiotic treatment comprised β-lactams (two with cloxacillin, two with ceftriaxone) and all were switched to co-trimoxazole or chloramphenicol based on susceptibility results.

Two isolates belonged to the international ST239-MRSA-III clone and two to ST59/952-MRSA-V(T) Taiwan Clone. One isolate each of ST2250-MRSA-IV (*S. argenteus*), ST2885-MRSA-V and CC398-MRSA-V was identified. The ST59/952-MRSA-V(T) Taiwan Clone and CC398-MRSA-V isolates encoded PVL and all three patients had documented abscesses. ST239-MRSA-III isolates demonstrated the highest diversity of antibiotic resistance and ST2250-MRSA-IV and ST2885-MRSA-V isolates the lowest.

### Virulence profiling.

56 (58%) isolates encoded PVL, including 38 of the 39 CC121 isolates. Other PVL-positive lineages were CC1 (8/12), CC88 (2/5), CC398 (5/5), ST59 (2/2), and ST2482 (1/1); half (28) were associated with abscesses; three patients also had positive blood cultures.

Toxic shock syndrome toxin-1 gene (*tst*) was detected in five isolates belonging to CC121, CC1, CC88, and ST1541. *Staphylococcus argenteus* lineages did not harbor detectable genes by microarray for capsule serotypes (*capH*, *capI*, *capJ, capK*) nor accessory gene regulator (*agr*) genes, as previously described.^16^ Exfoliative toxin A gene (*etA*) was uncommon and restricted to three lineages, CC121, ST2885, and CC188. Six of nine *etA*-positive isolates were recovered from children under five and one infant had positive blood cultures. Enterotoxin A gene (*sea*) was identified in 12 isolates (CC1, CC6, ST239), enterotoxin B gene (*seb*) in 41 isolates (CC121, CC1, CC188, ST59) and enterotoxin C gene (*sec*) in 10 isolates (CC1, CC88, ST1541). The enterotoxin gene cluster (*seg/i/m/n/o/u*) was present in all CC121, CC1223 and CC20 isolates. Arginine catabolic mobile element genes were not detected. Five immune evasion cluster (IEC) types were found and three isolates (CC121, ST2250, ST1541) were IEC negative.

Of the novel lineages identified, ST2885 isolates encoded *agrIV*, *cap5*, and *etA.* The ST1541 isolate harbored *agrIV*, *tst, cap8*, and *sec*. The virulence profiles for each lineage are detailed in Supplemental Table 2.

## DISCUSSION

This is the first comprehensive report of the molecular epidemiology of *S. aureus* SSTIs from Laos. Of note are the prominence of PVL-positive CC121 and other PVL-producing strains, the relatively low prevalence of MRSA, the presence of *S. argenteus* and the identification of two novel lineages. The globally widespread clones CC30 and CC5 were notably absent from this series despite their frequent presence in human carriage in Cambodia and worldwide.^9^

Due to resource limitations, we were only able to include a limited number of isolates and patients. Apart from basic demographic and clinical information, our study lacks details on clinical presentations and treatment outcomes and we did not have sufficient data to investigate potential epidemiological links. Despite these limitations, this report provides key baseline data about the characteristics of *S. aureus* SSTIs in Laos which will inform further exploratory studies and may be helpful to local clinicians.

CC121-MSSA dominated our series - this lineage is commonly found in SSTIs worldwide^17^ and is the main carriage strain in Cambodia.^9^ PVL genes were detected in most (97%) of our CC121 isolates and in keeping with recognized clinical characteristics of PVL-associated infection, half were associated with abscesses and occurred in young children. A significant proportion of isolates belonging to other lineages were also PVL positive. This relatively high prevalence of PVL genes compared with European countries^18^ is similar to northeast Thailand,^19^ New Zealand,^20^ and Africa.^21^ PVL-associated SSTIs can be recurrent in nature and may impose a significant burden on this population with limited healthcare resources. PVL has also been reported from patients with invasive disease and all of our bacteremic patients had PVL-positive isolates, although microbiologically-proven bacteremia was rare in our series. MRSA belonging to CC121 are uncommon^17^ and were absent from our series, although it has been described in pediatric SSTIs in Cambodia.^6^ In Laos, lack of access to antibiotics such as clindamycin and linezolid, commonly used for severe toxin-mediated infections in developed countries, needs to be addressed.

*S. argenteus* comprised 6% of the Lao samples and were recovered from older adults and those with diabetes, as previously observed.^10^ It has been suggested that *S. argenteus* is likely to cause a similar spectrum of disease to *S. aureus*^10^ but may be associated with a lower risk of respiratory failure in sepsis.^19^ These isolates may be misidentified as less pathogenic coagulase-negative staphylococcal species in laboratories in resource-poor settings because of the lack of the characteristic golden pigment. Both ST2250 and ST1223 have been found in neighboring countries, with the former accounting for the majority of invasive *S. argenteus* infections in Thailand.^19^ Our isolates were PVL-negative as described previously^8,10^ but it is worth noting that PVL was found in 16% of *S. argenteus* isolates in a recent Thai study of invasive disease.^19^ Only one of our isolates was methicillin-resistant, which is comparable to the situation in Thailand^19^ but contrasts with Australia where methicillin resistance is common in these lineages, highlighting their propensity to acquire *mecA*.^20^

The rarely reported ST2885 isolates encoded *cap5* which has been associated with invasive disease.^22^ MRSA within this lineage was recently described in a patient from Bangladesh with puerperal infection—like our isolates of this lineage, it was PVL-negative and *etA*-positive.^23^ The Bangladeshi isolate harbored SCC*mec* IVa in contrast to the Lao isolate, suggesting that the type V cassette had been acquired locally in Laos. The cluster of two infections we identified from a pediatric intensive care unit, supported by WGS data, may suggest cross-infection or a common external source of infection. The inferred phylogeny of ST2885 isolates suggests that the MRSA isolate, which sits within the MSSA clade, may have acquired methicillin resistance recently. This highlights the need for robust surveillance and infection control measures, which can be challenging to implement and maintain where staff and healthcare resources are overstretched. We did not find any other differences between neonates, infants and other age groups.

CC398, especially as MRSA, has gained notoriety as a livestock-associated lineage. Nevertheless, detailed genomic studies have shown that CC398 originated in humans^24^ and can cause SSTIs, bacteremia and necrotizing pneumonia.^25^ Although much initial interest in this lineage revolved around infections in pig farmers in Europe, China, and North America, ongoing transmission and infections independent of livestock exposure are now well-recognized.^26,27^ All five of our CC398 isolates (four MSSA, one MRSA) were PVL positive, *tetM*-negative and had detectable IEC genes, characteristics which suggest human rather than livestock origin.^26^ PVL-positive CC398-MRSA is endemic to Vietnam and China.^28,29^

Apart from penicillin and tetracycline resistance, our results suggest that there is currently little phenotypic or genotypic resistance to other antimicrobials amongst *S. aureus* in Laos. This information will help to inform local antibiotic policies. The proportion of MRSA in our series (7%) is lower than has been reported from surrounding countries: CA-MRSA rates of 30% in Vietnam and HA-MRSA rates of 57% in Thailand and 74% in Vietnam.^7^ We were unable to definitively classify infections as hospital- or community-acquired due to lack of clinical data but at least three of seven of our MRSA patients had healthcare-associated risk factors and one had likely travel-related acquisition. The MRSA lineages identified in our sample are circulating in other southeast Asian countries: ST239-MRSA-III is the dominant HA-MRSA strain in Thailand, Vietnam and Malaysia and ST59/952-MRSA-V(T) is a major CA-MRSA strain in Vietnam,^7^ suggesting spread from these countries and highlighting the potential for infiltration and dissemination within Laos. The dominant CA-MRSA strains in Cambodia, ST834-MRSA-IV and ST121-MRSA-V,^7^ were not present in our sample although MSSA belonging to these lineages were. Only 10 MRSA isolates were previously identified at the study laboratory over an 11-year period (2000–2011)^4^; the proportion of MRSA in this later series may possibly indicate an upward trend in MRSA rates in Laos, although increased sampling may also explain this difference. MRSA rates in Laos need to be further quantified and monitored. Glycopeptides and agents for topical eradication such as mupirocin and chlorhexidine are not available in Laos. Resistance to these agents was reassuringly absent in our series, apart from *qacA* in one MSSA isolate.

In summary, we have demonstrated both clear parallels in the molecular epidemiology of *S. aureus* between Laos and neighboring countries and interesting differences such as lower MRSA rates and the identification of several novel strains. The presence of regionally circulating epidemic MRSA clones in our sample suggests infiltration of these lineages from surrounding countries into Laos and warrants expanded vigilance.

## Supplementary Material

Supplemental Table.
