# Supplementary material for: Molecular Epidemiology of Staphylococcus aureus Skin and Soft Tissue Infections in the Lao People’s Democratic Republic
Source: Am J Trop Med Hyg. 2017 May 30;97(2):423–8. doi: 10.4269/ajtmh.16-0746 (PMC5544078; doi:10.4269/ajtmh.16-0746)
Supplement: Supplementary file 1 [file tpmd160746.SD1.pdf]

Supplemental Table 1

Staphylococcal protein A gene (*spa*) types by lineage for all isolates (*N* = 96)

Lineage	*spa* type	No. of isolates
CC121-MSSA	t159	18
	t7002	10
	t3204	6
	t4499	2
	t272	1
	t610	1
	t645	1
CC1-MSSA	t127	4
	t114	1
	t3963	1
	t5445	1
	t14829	1
	t14830	1
	t14832	1
	t14833	1
	t14834	1
CC6-MSSA	t701	5
	t304	1
ST2250-MSSA	t5787	2
	t6675	2
ST2250-MRSA-IV	t7960	1
CC1223-MSSA	Non-typeable*	1
ST2885-MSSA	t13849	4
	t14831	1
ST2885-MRSA-V	t13849	1
CC88-MSSA	t186	1
	t1376	1
	t4494	1
	t13981	1
	t14043	1
CC398-MSSA	t034	4
CC398-MRSA-V	t034	1
CC188-MSSA	t2883	2
	t189	1
	t13969	1
CC97-MSSA	t267	1
	t521	1
	t11511	1
CC20-MSSA	t2919	1
	Non-typeable†	1
ST239-MRSA-III	t4835	1
	t4854	1
ST59/952-MRSA-V(T)	t437	2
CC15-MSSA	t774	1
ST834-MSSA	t213	1
ST2482-MSSA	t9805	1
ST1541-MSSA	t13785	1

spa = staphylococcal protein A gene; CC = clonal complex; ST = multilocus sequence type; MSSA = methicillin-sensitive *Staphylococcus aureus*; MRSA = methicillin-resistant *S. aureus*.

* A *spa* type could not be assigned by the Ridom *spa* server because of a sequence polymorphism in the consensus region.

† A *spa* type could not be assigned by the Ridom *spa* server because of the insertion of irregular-sized repeat sequences.

Supplemental Table 2

Virulence markers by lineage for all isolates ($N = 96$)

	Total	PVL	PVL + abscess	*tst*	*cap5*	*cap8*	*etA*	*etB*	*sea*	*seb*	*sec/l*	*sed*	*seh*	*sek/q*	*egc*	*agrI*	*agrII*	*agrIII*	*agrIV*	*agr-*	*scn*	*scn + sak*	*scn+ sak+ sea*	*scn+ chp*	*scn+ chp+ sak*	IEC-
CC121-MSSA	39	38	17	2		39	2	1		37		1			39				39			38				1
CC1-MSSA	12	8	4	1		12			4	1	8		12	9				12			8		4			
CC6-MSSA	6					6			6					4		6							6			
ST2250-MSSA	4																			4		3				1
CC1223-MSSA	1														1					1	1					
ST2885-MSSA	5				5		5												5			5				
CC88-MSSA	5	2		1		5					1							5				2			3	
CC398-MSSA	4	4	3		4							1				4									4	
CC188-MSSA	4					4	1			1						4						3			1	
CC97-MSSA	3				3											3						3				
CC20-MSSA	2				2										2	2									2	
CC15-MSSA	1					1											1							1		
ST834-MSSA	1					1										1						1				
ST2482-MSSA	1	1	1		1												1					1				
ST1541-MSSA	1			1		1					1								1							1
ST239-MRSA-III	2					2			2					2		2							2			
ST59/952-MRSA-V(T)	2	2	2			2				2				2		2								2		
ST2250-MRSA-IV	1																			1		1				
ST2885-MRSA-V	1				1		1												1			1				
CC398-MRSA-V	1	1	1		1											1									1	
Total	96	56	28	5	17	73	9	1	12	41	10	2	12	17	42	25	2	17	46	6	9	58	12	3	11	3

CC = clonal complex; ST = multilocus sequence type; MSSA = methicillin-sensitive *Staphylococcus aureus*; MRSA = methicillin-resistant *S. aureus*; PVL = Panton-Valentine leukocidin; PVL + abscess = Panton-Valentine leukocidin genes detected and abscesses documented for clinical presentation; *tst* = toxic shock syndrome toxin-1 gene; *cap5/8* = capsule serotype 5 or 8 genes; *etA/B* = exfoliative toxin a or b genes; *sea/b/c/d/h/k/l/q* = staphylococcal enterotoxin a/b/c/d/h/k/l/q genes; *egc* = enterotoxin gene cluster (*seg/i/m/n/o/u*); *agrI-IV* = accessory gene regulator groups I-IV genes; agr- = accessory gene regulator genes not detected; *scn* = staphylococcal complement inhibitor gene; *sak* = staphylokinase gene; *chp* = chemotaxis inhibiting protein gene; IEC- = immune evasion cluster genes (*scn*, *sak*, *chp, sea*) not detected.
